# Proximity to Pollution Sources and Risk of Amphibian Limb Malformation

**DOI:** 10.1289/ehp.7585

**Published:** 2005-07-11

**Authors:** Brynn Taylor, David Skelly, Livia K. Demarchis, Martin D. Slade, Deron Galusha, Peter M. Rabinowitz

**Affiliations:** 1 School of Forestry and Environmental Studies, Yale University, New Haven, Connecticut, USA; 2 Occupational and Environmental Medicine Program and; 3 Department of Epidemiology and Public Health, Yale University School of Medicine, New Haven, Connecticut, USA

**Keywords:** agriculture, amphibian, animal sentinel, malformation, teratogen, water pollution

## Abstract

The cause of limb deformities in wild amphibian populations remains unclear, even though the apparent increase in prevalence of this condition may have implications for human health. Few studies have simultaneously assessed the effect of multiple exposures on the risk of limb deformities. In a cross-sectional survey of 5,264 hylid and ranid metamorphs in 42 Vermont wetlands, we assessed independent risk factors for nontraumatic limb malformation. The rate of nontraumatic limb malformation varied by location from 0 to 10.2%. Analysis of a subsample did not demonstrate any evidence of infection with the parasite *Ribeiroia*. We used geographic information system (GIS) land-use/land-cover data to validate field observations of land use in the proximity of study wetlands. In a multiple logistic regression model that included land use as well as developmental stage, genus, and water-quality measures, proximity to agricultural land use was associated with an increased risk of limb malformation (odds ratio = 2.26; 95% confidence interval, 1.42–3.58; *p* < 0.001). The overall discriminant power of the statistical model was high (*C* = 0.79). These findings from one of the largest systematic surveys to date provide support for the role of chemical toxicants in the development of amphibian limb malformation and demonstrate the value of an epidemiologic approach to this problem.

In 1995, amphibians with severely malformed limbs were discovered in Minnesota (Blaustein and Johnson 2003). The next year, amphibians with truncated and missing limbs were found at several sites stretching along 120 miles of Vermont’s Lake Champlain shoreline (Levey et al. 2003). Since then, elevated rates of developmental abnormalities have been found in most U.S. states and Canada, raising concern that amphibians are serving as animal sentinels of human environmental health hazards (Burkhart et al. 1998, 2000; Daszak et al. 2001; van der Schalie et al. 1999).

The exact etiology of amphibian limb abnormalities (including missing, extra, and malformed limbs) remains unclear. The major suspects have been ultraviolet B (UV-B) radiation, trauma, parasitic trematode infestation, and xenobiotic pesticides or chemicals (Ouellet 2000). Although many researchers have searched for single causative agents, some have stated that the etiology is likely multifactorial (Linder et al. 2001).

In laboratory settings, some evidence has supported each of the etiologic hypotheses. Controlled exposure to UV radiation has produced amphibian dysmorphogenesis (Ankley et al. 1998, 2002) and truncated limbs (Levey et al. 2003; Meteyer et al. 2000; Ouellet et al. 1997). These studies, however, have generally failed to reproduce the spectrum of abnormalities occurring in wild populations. The abnormalities that result from experimental UV exposures are most often bilateral and symmetrical (Ankley et al. 1998, 2002), whereas abnormalities found in the wild are not (Levey et al. 2003; Meteyer et al. 2000; Ouellet et al 1997). Furthermore, the levels of UV exposure used in the laboratory experiments may not reflect real-world exposures.

Another primary etiologic hypothesis of limb abnormality is trauma related to predation. Although predation can account for some forms of abnormality in the wild, the hypothesis, more generally, has not been supported as the primary cause of increased amphibian abnormality rates in recent years (Blaustein and Johnson 2003; Levey et al. 2003). Although predation should always be considered in this field of research, it is likely that deformities caused by predation have a different etiologic pathway and therefore, in research, should be distinguished from the other hypotheses of dysmorphogenesis.

Macroparasite infection is the third etiologic hypothesis of amphibian limb abnormality. The parasite hypothesis continues to receive substantial attention and is perhaps the most thoroughly explored of all the hypotheses (Blaustein and Johnson 2003). The most commonly explored parasite is the trematode *Ribeiroia ondatrae*. Infestation with *Ribeiroia* is associated with limb abnormalities in some amphibian species (Johnson et al. 2001, 2002). Laboratory experiments suggest that the mechanism of *Ribeiroia*-induced abnormalities may involve mechanical disturbance of growing limb cells or interference with a retinoid-sensitive signaling pathway (Johnson and Sutherland 2003).

The role of exposure to potential chemical teratogens has also been investigated. Candidate toxicants have included nonpolar organics, metals (Burkhart et al. 1998; Linder et al. 2001; Stocum 2000), herbicides, pesticides, and other components of agricultural runoff (Bishop et al. 1999; Ouellet et al. 1997), sewage (Linder et al. 2001; Ouellet 2000), and pharmaceuticals (Mizgireuv et al. 1984). Such chemical agents could directly affect amphibians’ development or act indirectly by increasing amphibian susceptibility to other environmental stressors such as infectious disease, predation, and UV-B light. Investigations into chemically mediated limb abnormalities have used two major approaches. First, amphibian eggs and larvae have been exposed to water and sediment collected from field sites with high abnormality rates (Fort et al. 1999, 2001). Second, the specific suspected amphibian teratogens have been tested in the laboratory using toxicologic assays (Degitz et al. 2000, 2003; LaClair et al. 1998). Together, these studies have shown that exposure to field-collected water and sediment can result in limb abnormalities and that a number of chemicals can have severe teratogenic effects on amphibians related to dose or concentration (Burkhart et al. 1998). However, there are inconsistencies between laboratory and field results and no single causative chemical has been identified.

The chemical teratogen hypothesis has particular relevance to human health risk. If waterborne chemical toxicants are involved in amphibian malformations, there is potential for shared exposure with human populations through dermal contact, ingestion, and inhalation routes.

Despite the fact that multiple hypotheses have emerged to explain the phenomenon of amphibian limb abnormalities, few studies to date have made use of research techniques allowing examination of multiple factors. For example, most studies of chemical causes did not also collect data regarding infectious agents, and few of the studies have used multivariable statistical techniques to model the relative effects of different factors.

We performed a cross-sectional study of risk factors for deformities in a large systematic sample of amphibians. The large sample size allowed for the creation of a multivariate model to test the association between amphibian limb malformation and a number of independent risk factors. The types of land use adjacent to wetlands where amphibians were surveyed served as a proxy measure for possible sources of water pollution. We hypothesized that, after adjusting for confounding factors, amphibians in wetlands adjacent to agriculture, septic systems, or lawns would be at greater risk for amphibian hind limb malformation due to the waterborne chemical runoff associated with such land uses.

## Materials and Methods

### Study sample.

Between 24 May and 28 August 2002, amphibian specimens consisting of two species of hylids (*Hyla versicolor* and *Pseudacris crucifer*) and four species of ranids (*Rana pipiens*, *Rana catesbeiana*, *Rana clamitans*, and *Rana sylvatica*) were collected from 42 wetlands in the Lake Champlain Basin of Vermont. We selected wetlands in a representative fashion along an urbanization gradient ranging from relatively undisturbed forest habitat (Green Mountain National Forest), to rural communities, to neighborhoods in Burlington. To collect specimens at each site, standardized collection methodology consisted of pipe samplers (steel pipe, 35-cm diameter, 0.91 m long) and dip nets (46 cm × 23 cm with 1-mm mesh). An attempt was made to collect up to 300 specimens from each site (a maximum of 100 during each of three visits). After capture, specimens were first anesthetized in a solution of tricaine methanesulfonate and then placed in 70% ethanol, according to protocols for the study that were approved by the Yale University Animal Care and Use Committee. Each specimen was later examined in the laboratory in order to determine genus and species, Gosner stage, and presence of malformation. A subsample of specimens was examined by dissection for the presence of parasite metacercariae, including *Ribeiroia*.

### Exposure assessment.

For each of the 42 wetlands, on-site field observations were used to determine whether agriculture or lawns were located proximate to the wetland. The presence of agriculture and lawns were then scored as yes/no responses for each site. In addition, we independently estimated land use/land cover within 200 m of each amphibian sample site, which was calculated using geographic information system (GIS) data. The latitude and longitude coordinates of each site were used with land-use/land-cover GIS layers to map the land use within 200 m of each site. The Raster land-cover data set from Vermont GIS (U.S. Geological Survey 1999) was brought into ArcMap (Environmental Systems Research Institute, Redlands, CA) and was converted to vector data (polygons) to allow clipping of the features for each buffer zone. These polygons were then clipped using the geoprocessing command by a 200 m buffer surrounding the GPS (global positioning satellite) coordinates representing the study site’s general area. These clipped land-use/land-cover areas were then summarized for each location by their classification codes to calculate land-cover percentages represented in the data. For this analysis, the 21 possible land-use categories were used to determine the presence of agriculture or forest. We compared the GIS measures for agriculture and forest with field observations of agriculture and lawns. There was a strong correlation between GIS coded agriculture land-use and field observations of agriculture and lawn near the study site (*p* < 0.0001 for both). Both observed agriculture and lawns were negatively correlated with GIS land-use estimates for forest cover (*p* < 0.0001 for both).

Several quantitative measurements were made at each site, including pH, conductivity, dissolved oxygen, temperature, total nitrogen, and total phosphorus for the water samples. If more than one measurement took place at an individual wetland, the measurements were averaged together for that site.

As an additional investigation into the possible role of parasitic infection, a representative sample of snails from the study wetlands was investigated for evidence of *Ribeiroia* infection.

### Case definition.

Each individual specimen was examined by a trained technician and classified according to a case definition adapted from a well-established amphibian limb abnormality classification scheme (Meteyer et al. 2000). To be classified as a “case” of limb malformation, a specimen had to exhibit one of the following: missing or reduced hind limb elements, complete but malformed hind limb, and/or duplicated hind limb elements or segments.

For the analysis of limb malformations, we included only those specimens with Gosner stage of ≥26 (Gosner 1960). At this stage, hind limbs are visible, and it is possible to assess whether gross deformities are present.

Of the original 5,983 specimens, this led to the exclusion of 684 with stage < 26, and 27 with missing stage, leaving 5,272. An additional 8 cases were excluded because of unknown length, leaving 5,264 in the final study sample. For our case definition, we excluded trauma deformities (abnormalities that appeared to be a result of trauma, including presence of open wounds, edema, scarring, bone fractures, etc.) because of the differing etiologies of trauma and nontrauma abnormalities.

### Statistical analysis.

All analyses were performed using SAS software (version 8.02; SAS Institute Inc., Cary, NC).

Simple frequencies were calculated for categorical variables, including Gosner stage, genus, water-quality measures, presence of pollution sources, and presence of limb malformation. Means and SDs were calculated for continuous variables.

To determine bivariate associations between exposures and rates of malformation, we performed simple logistic regression. Odds ratios (ORs) and 95% confidence intervals (CIs) were calculated for each individual factor. An OR calculates the odds of a risk factor being present in the affected cases compared with the odds that it is present in the non-affected individuals. A multivariate logistic regression model was developed to determine the independent predictors of malformation. To determine which variables to include in the multivariate model, we examined collinearity between independent variables using correlation coefficients. Variables with correlations above levels at which multicollinearity would be considered likely (*r* > 0.40) were excluded from the multivariable analyses. We found that a number of variables had significant collinearity, including nitrogen and phosphorus (*r* = 0.67) and pH and dissolved oxygen (*r* = 0.79). Therefore, neither phosphorus nor pH was included in the final multivariate model.

The multivariate logistic regression analysis used a backward selection process to eliminate nonsignificant variables from the model (criteria for elimination from the model set at *p* > 0.05). ORs, 95% CIs, and *p*-values for association between individual risk factors and limb malformation were determined for variables remaining in the final model. The overall discriminant power of the model was assessed using a *C*-statistic.

## Results

### Characteristics of study sample.

Table 1 shows the characteristics of 5,264 specimens meeting eligibility criteria (specimens with Gosner stage of ≥26). The average Gosner stage was 36 (field stage 4: toes 3–5 separated). Overall, 83 specimens (1.6%) showed evidence of non-traumatic limb malformation. The study-site–specific rate of malformation ranged from 0 to 10.2%. The lowest rates were found in the wetlands located in the Green Mountain National Forest. Table 1 also shows the relative prevalence of subtypes of nontraumatic malformation. The most common type was malformed limb or element (68 of 83), followed by missing limb or element (25 of 83). Some individuals had more than one type of abnormality.

Examination of a subsample of individual specimens (*n* = 40) revealed no evidence of *Ribeiroia* infection. Similarly, representative samples of host snails from the sample wetlands did not demonstrate evidence of *Ribeiroia* infection (Skelly D, unpublished data).

### Exposure assessment.

Table 2 summarizes the exposure characteristics of the 42 wetland study sites. More than 40% of the wetlands had some degree of agriculture nearby; this ranged from pastureland to cropland to intensive dairy farming. Lawns were present near > 35% of wetlands. The wide range of values for water-quality measures reflects, in part, variations in runoff sources; the wide variability of conductivity reflects proximity to roads treated with salt in winter. The highest nitrogen readings were found in a pond located downhill from a dairy barn.

### Risk factor analysis.

Table 3 shows the simple and multiple logistic regression measures of association between different risk factors and nontraumatic amphibian limb malformation. In the simple (bivariate) analyses, Gosner stage, proximity to agriculture, proximity to lawns, and dissolved oxygen each showed a positive association with malformation.

In the multivariate logistic regression model, Gosner stage remained highly significantly associated with malformation (OR = 1.18; *p* < 0.0001). In other words, the risk of malformation increased 18% for each increase in Gosner stage. Although malformation rates were slightly higher in ranids than in hylids, the effect of genus was not significant in the multivariate model. Furthermore, analyzing risk factors for malformation in ranid species alone or hylid species alone produced results similar to those of our combined model (data not shown). In the multivariate model, proximity to agriculture remained a highly significant predictor of malformation risk (OR = 2.26; 95% CI, 1.42–3.58; *p* < 0.001). None of the other independent variables tested showed a significant association in the multivariate model. The discrimination and fit of the logistic regression model were good, with a *C*-statistic = 0.79 and a *p*-value for the Hosmer-Lemeshow goodness-of-fit test of 0.10.

## Discussion

The results of this study, based on one of the largest systematic sampling of limb deformities in wild amphibian populations to date, suggest that the composition of landscapes surrounding wetlands affects rates of limb malformation. In particular, proximity to human-associated land uses, including agriculture and lawns, is associated with an increased risk. This positive association persists even after adjusting for the effect of developmental stage and variation in water-quality measures such as nitrogen and pH. Proximity to agriculture was associated with a more than doubling of the risk of limb malformation.

Major strengths of this study include its sample size and systematic design. Although other studies have assessed agricultural land use in relation to amphibian population size in specific geographic areas (Davidson et al. 2001, 2002; Ray et al. 2002), few studies have assigned exposure variables based on observed pollution sources in a systematic manner in order to assess malformation rates. Several studies have discussed land use and land-use change as drivers of the abnormality and decline phenomena (Collins and Storfer 2003; McCallum and Trauth 2003) but have not gone on to analyze outcomes with respect to these factors.

Unlike many previous studies, this study uses a human epidemiologic methodology to assess an ecologic problem (with possible relevance to human health). Evaluation of multiple stressors will require continued development of effective study methodologies and a movement away from the single-agent hypothesis testing of the past (Rabinowitz et al. 1999). To our knowledge, this is one of the first studies to use multivariate techniques (which require a large sample size) to evaluate the relative importance of multiple factors while adjusting for possible confounding. This reduces the possibility that an observed association between a risk factor (e.g., agriculture) and the outcome of interest (limb malformation) is due in fact to a confounding risk factor that is linked with both the exposure and the outcome in such a way that spurious etiologic associations are inferred.

What is the possible biologic basis of the observation that proximity to agricultural land use is associated with risk for amphibian limb malformation? We argue that exposure to sources of anthropogenic pollution is a likely explanation. Without identification of specific compounds, any specific hypothesis remains speculative. However, agricultural runoff may include a variety of chemicals, including pesticides, animal wastes, and fertilizers, that could threaten water quality. One study of an agricultural watershed found evidence of 38 pesticides in water samples, including 30 herbicides, 4 fungicides, 3 insecticides, and 1 metabolite of one of the herbicides (Kreuger 1998). Water-borne toxicants in agricultural runoff could directly affect development, either singly or in chemical mixtures (Burkhart et al. 2000). Agricultural runoff has been associated with impaired hatching success (De et al. 2002). Other lipophilic chemicals in runoff may also play a role—recent studies found that exposing *Rana pipiens* tadpoles to extracted compounds from semipermeable membrane devices in freshwater ponds could cause malformation in the presence of UV radiation (Bridges et al. 2004).

There are a number of biologic mechanisms by which chemicals in agricultural runoff could cause deformities. A recent study found an association between agricultural runoff including the herbicides atrazine, deethyl-atrazine, simazine, metolachlor, dimethenamide, chlopyralide, dicamba, and bentazone and plasma retinoid levels in *Rana catesbiana* (Berube et al. 2005); retinoids appear to function in signaling pathways for limb development (Stocum 2000). It is also possible that herbicides or other organic compounds could directly affect the expression of genes that help determine limb development. Because thyroid hormone has been found to protect against the development of abnormalities in the experimental setting (Fort et al. 2001), chemically induced alterations of thyroid function could potentially increase the malformation rate.

Another potential mechanism for the effect of agricultural chemicals on amphibian malformation could be through effects on either parasite population density or on host immune function leading to increased rate of parasite infection (Christin et al. 2003). Our investigation considered the possibility that parasitic infection with *Ribeiroia* could have played a role in the etiology of limb deformities at the study sites. However, our analysis to date does not support the parasitic infection hypothesis. Examination of individual amphibians as well as representative samples of host snails from the study sites did not reveal evidence that either this parasite or trematode infection in general was playing a role in the causation of limb malformation.

Some studies have suggested that nutrient loads such as phosphorus and nitrogen, present in some agricultural runoff, could contribute to developmental abnormality risk by contributing to eutrophication (Johnson and Chase 2004). In this sample, we had the opportunity to adjust for the effect of measured nutrients in sampled water and found that these nutrients did not remain significant predictors of malformation in the multivariate model.

There are several possible reasons why the overall rate of nontraumatic hind limb abnormality in this study sample (1.6%) was lower than figures reported for amphibian limb abnormalities in some other surveys (Burkhart et al. 2000). First, there was variation in rates of malformations between study sites, with rates in excess of 10% at some sites. The systematic nature of the present survey was designed to reduce selection bias, and most study sites were consequently selected without prior knowledge of the site-specific rate of amphibian limb abnormalities. As a result, in some locations, no abnormalities were detected. Second, we used a more restrictive case definition than many other surveys, taking care to exclude trauma-induced limb deformities, thereby lowering the overall abnormality rate. Third, rates of abnormalities fluctuate from year to year in individual wetlands, and some of the ponds sampled had experienced abnormality rates as high as 30% in previous years (Levey et al. 2003). It therefore seems likely that the variability in malformation rates between ponds was related to environmental factors.

The study relied on a limited number of measurements of water quality, as well as visual observations of land use as an indicator of potential pollution sources. Therefore, the observed associations between runoff sources, measured water quality, and malformation rate must be viewed as preliminary. Determining exposure at the level of the group rather than the individual risks the “ecologic fallacy” of assigning potential causation to one factor when in fact another, unmeasured factor is actually responsible. Although the rate of malformation in ponds near agriculture was increased, it could be that this difference was due to other factors that were not assessed. Further studies involving direct measurements of toxicants in water, and case–control comparisons of affected and unaffected individuals need to be carried out in order to identify candidate toxicants or chemical mixtures that could be investigated in the laboratory setting.

Water-quality measures are dependent on multiple factors and vary over a season and year to year depending on runoff sources, rainfall, temperature, and other factors. By using a limited number of water-quality measures taken during a single year, it is possible that some exposure misclassification occurred, with water conditions during sensitive periods of amphibian development varying from the measurements at the time of sampling. However, such misclassification, in epidemiologic studies of cause and effect, tends to be nondifferential and bias the results toward observing no effect (Checkoway and Eisen 2005). Therefore, the fact that we found strong associations between a number of exposures and the rate of malformation indicates that such associations are real and significant. The good discrimination and fit of the multivariate logistic regression model further support the strength of the observed associations.

The human health relevance of these findings remains to be determined. If a chemical toxicant or mixture could be identified that is capable of causing limb malformations in wild amphibians, several additional lines of investigation could shed light on whether a risk to humans exists. First, it would be useful to know what human health outcome would be analogous to amphibian limb deformities; this could be explored if gene sequence homology between humans and amphibians was present for target genes affected by the toxicants. Second, it would be important to establish a dose–response relationship and determine whether environmental exposures to such a toxicant would be capable of causing human health effects. Finally, epidemiologic studies including both amphibian and human populations could determine whether rates of malformation in amphibians show a correlation with a suspected human health outcome. Although to date none of these connections have been established, they will be worthy of further exploration if confirmatory studies support the role of toxicant chemicals in the etiology of amphibian developmental abnormalities.

## Figures and Tables

**Table 1:** Characteristics of the study population (*n* = 5,264).

				Malformation type^a^		
Species	No. (%)	Gosner stage (mean ± SD)	Malformation [*n* (%)]	Missing	Malformed	Extra
*Hyla versicolor*	235 (4.5)	36.0 ± 6.4	4 (1.7)	1	3	0
*Pseudacris crucifer*	895 (17.0)	34.8 ± 6.2	6 (0.7)	3	3	0
Total hylids	1,130 (21.5)	35.0 ± 6.2	10 (0.9)	4	6	0
*Rana catesbeiana*	319 (6.1)	37.7 ± 6.7	5 (1.6)	2	3	0
*Rana clamitans*	1,176 (22.3)	40.3 ± 6.2	32 (2.7)	9	28	2
*Rana pipiens*	1,702 (32.3)	35.8 ± 8.0	33 (1.9)	10	28	0
*Rana sylvatica*	937 (17.8)	31.7 ± 5.2	3 (0.3)	0	3	0
Total ranids	4,134 (78.5)	36.3 ± 7.5	73 (1.8)	21	62	2
Total	5,264 (100)	36.0 ± 7.2	83 (1.6)	25	68	2

a Some individuals exhibited more than one type of malformed limb or element.

**Table 2:** Exposure assessment for study sites (*n* = 42).

Characteristic	Exposure
Proximity to pollution sources [*n* (%)]	
Agriculture nearby or adjacent	17 (40.5)
Lawn nearby or adjacent	15 (35.7)
Water-quality measures	
pH	7.6 ± 0.9 (5.7–9.7)
Conductivity (μS)	303.3 ± 307.8 (10–1,350)
Dissolved oxygen (mg/L)	6.5 ± 3.4 (1.2–18.3)
Temperature (°C)	21.2 ± 3.6 (12.8–29.1)
Total nitrogen (mg/L)	0.8 ± 0.8 (0.1–5.4)
Total phosphorus (mg/L)	0.2 ± 0.4 (0.004–2.2)

μS, micro-Siemens. Values shown are mean ± SD (range) except where indicated.

**Table 3:** Risk factors for nontraumatic limb malformation.

	Bivariate analysis		Multivariate model^a^	
Risk factor	OR (95% CI)	*p*-Value	OR (95% CI)	*p*-Value
Characteristic				
Gosner stage	1.20 (1.14–1.26)	< 0.0001	1.18 (1.13–1.24)	< 0.0001
Genus (*Rana* vs. *Hyla*)	2.01 (1.04–3.91)	0.04	—	—
Proximity to pollution sources				
Agriculture (nearby or adjacent)	3.08 (1.96–4.85)	< 0.0001	2.26 (1.42–3.58)	< 0.001
Septic system or lawn adjacent	2.06 (1.34–3.19)	< 0.001	—	—
Water-quality measures				
Conductivity (μS)	1.00 (1.00–1.00)	0.46	—	—
Dissolved oxygen (mg/L)	1.14 (1.06–1.23)	< 0.001	—	—
Temperature (°C)	1.05 (0.98–1.13)	0.14	—	—
Total nitrogen (mg/L)	1.22 (0.91–1.63)	0.18	—	—

μS, micro-Siemens.

a Certain variables were removed from the model during backward selection modeling (—; exclusion criteria, *p* > 0.05).
